# Long distance travelling and financial burdens discourage tuberculosis DOTs treatment initiation and compliance in Ethiopia: a qualitative study

**DOI:** 10.1186/1471-2458-13-424

**Published:** 2013-05-01

**Authors:** Takele Tadesse, Meaza Demissie, Yemane Berhane, Yigzaw Kebede, Markos Abebe

**Affiliations:** 1 Institute of Public Health, the University of Gondar, Gondar, Ethiopia; 2 Addis Continental Institute of Public Health, Addis Ababa, Ethiopia; 3 Armauer Hansen Research Institute, Addis Ababa, Ethiopia

## Abstract

**Background:**

Timely tuberculosis treatment initiation and compliance are the two key factors for a successful tuberculosis control program. However, studies to understand patents’ perspective on tuberculosis treatment initiation and compliance have been limited in Ethiopia. The aim of this study is to attempt to do that in rural Ethiopia.

**Methods:**

This qualitative, phenomenological study conducted 26 in-depth interviews with tuberculosis patients. A thematic content analysis of the interviews was performed using the Open Code software version 3.1.

**Results:**

We found that lack of geographic access to health facilities, financial burdens, use of traditional healing systems and delay in diagnosis by health care providers were the main reasons for not initiating tuberculosis treatment timely. Lack of geographic access to health facilities, financial burdens, quality of health services provided and social support were also identified as the main reasons for failing to fully comply with tuberculosis treatments.

**Conclusions:**

This study highlighted complexities surrounding tuberculosis control efforts in Dabat District. Challenges of geographic access to health care facilities and financial burdens were factors that most influenced timely tuberculosis treatment initiation and compliance. Decentralization of tuberculosis diagnosis and treatment services to peripheral health facilities, including health posts is of vital importance to make progress toward achieving tuberculosis control targets in Ethiopia.

## Background

The National Tuberculosis Control Program of Ethiopia faces many challenges despite its significant achievements in case detection and treatment success. The country still ranks 7^th^ in terms of TB burden worldwide and needs to progress towards the Stop TB Partnership targets of halving prevalence and deaths from TB by 2015 [1]. The key challenges against attaining these targets are delay in diagnosis and non-compliance to treatment by TB patients. These may increase the risk of the spread of infection in the community, threaten the success of treatment, increase the risk of multidrug resistance, as well as relapse, and death [2-6].

Timely treatment initiation and compliance are the two central issues to the success of TB control programs [7]. In Ethiopia, where there are increasing incidences of new infectious TB cases [8], quantitative studies from urban and rural areas found that the mean delay between the onset of TB symptoms and initiation of treatment ranges from 13 to 20 weeks [9,10] which is higher than the 4 week cut-off point commonly used to define long delays [11]. The rate of default from TB treatment ranges from 12 to 20% [12,13] which is also higher than the World Health Organization recommendation of less than 10% [14].

Evidence from a variety of literature shows that there are many factors affecting timely TB treatment initiation and compliance [15-26]. However, in Ethiopia, only a few studies looked into these factors and reported them quantitatively without further exploration into the issue owing particularly to the underutilization of qualitative research. The aim of this study was thus to explore reasons for TB treatment initiation and compliance from the lived experience and views of TB patients in Dabat district, Ethiopia.

## Methods

A qualitative, phenomenological study was carried out at the Dabat Health and Demographic Surveillance System site (HDSSs), owned by the University of Gondar, from July to December, 2011. The site is located in a district known as Dabat, northwest Ethiopia and has an estimated population of 46,165 living in 7 rural and 3 urban "kebeles" (the smallest administrative units in Ethiopia).Information on vital events, like birth, death, and migration were collected quarterly. The district has two health centers, three health stations, and twenty-nine health posts providing health services to the community. Only the two health centers in the study area provide Directly Observed Treatment Short Course (DOTS) to TB cases [27]. The DOTS coverage of the district by government health facilities is about 95%. TB treatment involves daily attendance for two months (the intensive phase), followed by a period of four months during which patients collect drugs weekly (the continuation phase). At the time of the study, HIV counselling and testing was routinely carried out for TB patients [28].

### Participants and data collection

A TB Surveillance Project based on active and passive case findings at the Dabat HDSSs [10] identified 137 smear-positive TB patients aged ≥14 years between 1 October, 2010 and 30 September, 2011. The study included TB patients who were attending treatment and those who had interrupted treatment. Newly diagnosed TB patients and patients who had interrupted treatment were approached by means of invitation letters distributed by the health personnel working at DOTS clinics in the study area. A purposive sample of 26 patients, fifteen women and eleven men aged 18 to 50, participated in the study. To ensure reasonable spread, range of participants with respect to age, sex, and residence were selected. Individuals who were seriously sick and mentally disabled excluded.

A trained research assistant (RA) performed the one-to-one in-depth interviews using a semi-structured guide in venues convenient for the participants. The guide was developed in English and translated into Amharic (the local language) for all participants. Interviewees narrated their live experiences from both individual and health system level perspectives. They were interviewed in their own houses, and access was ensured through TB surveillance project field workers at the Dabat HDSS site. Interviews lasted 40 to 60 minutes and were audio-taped with the consent of participants. The RA was guided by participant responses in deciding when and how to probe the emergent themes. Interviews continued until all categories were well-defined and saturated after interviewing the twenty-six participants. The principal investigator and the research assistant selected the research participants, closely monitored the in-depth interview process, took field notes and had regular meetings with the RA.

Qualitative content analysis of the interviews was performed using the Open Code software version 3.1. The RA transcribed and translated each interview. The translated material was read several times in order to get the general sense of the content. An inductive approach was followed to allow conceptual clustering of ideas and patterns to emerge. This process included a descriptive phase of identifying meaning units and assigning codes which were then compared and reorganized into tentative categories.

### Ethical considerations

The study protocol was reviewed and approved by the Institutional Review Board of the University of Gondar. Written consent was obtained prior to each interview and interviewee anonymity was guaranteed.

## Results

### Characteristics of the study population

A total of twenty-six participants were successfully interviewed, of which 15 were female and 11 male. The mean age of the participants was 35 years (range 18 to 50 years). About 28% were married and 8% had secondary school qualifications, grades 9 to12. Thirteen participants were not employed. Most of the study participants were rural dwellers. Two main themes which emerged from the content analysis are reported in detail below.

### Theme one: reasons for delay in tuberculosis diagnosis

#### Geographic access to health facilities

The main reason for delay in seeking TB treatment initiation was patients’ lack of physical access to health facilities which providing diagnostic and treatment services. The majority of the TB patients come from rural areas which were not within reach of a health facility in a two-hour walk. Furthermore, patients were required to travel long distance which was characterized by natural barriers, such as mountains and rivers. The effect of distance on TB treatment initiation was narrated as follows by a male patient aged 23 years:

“Well, I didn’t come to the health center early because it is far from my village …but now I am on TB treatment and I will just continue my treatment."

"Many patients from rural areas do not come to the health facility in time. They come here when they are seriously sick. They face more problems even after they are diagnosed as TB patients and their prognosis is very poor," says a 26 year-old male patient.

#### Financial burden

We found that the main reason for delayed treatment initiation was patients’ limited access to finance. Patients need money for transportation to health facilities, registration at health facilities, diagnosis, drugs, expensive referrals to the nearby hospitals and to meet other basic needs. Financial constraints led to considerable time loss before obtaining a diagnosis and appropriate treatment. What a 47-year-old male patient stated confirms this:

*"I, myself decided much early to go to Woken Health Center, which is one of the public health facilities in the study area. I knew some health professionals there… but I could not do it early enough due to lack of money for transportation."* A 22-year-old female patient also said: *"I was told to go for an X-ray to a hospital which is 25 km from the study health center …due to lack of money for travel and diagnosis I stayed for a while before going for the test."*

#### Use of traditional healing system

The majority of the TB patients reported about their use of traditional healing systems before going to health care facilities. They reported that the treatments were effective, helpful and kept away evil spirits. After the onset of TB symptoms, most patients applied self-medication using traditional medicine before seeking help from health care providers, and they went to health facilities when their symptoms persisted for some time and their health deteriorated. A 23- year- old male TB patient reported his use of an alternate healing system hereunder:

*"I use it because the treatment is effective and helpful. The extra thing with the holy water I get from the spiritual healer is that it helps in keeping away evil spirits."* Another 25-year-old female participant diagnosed with TB three months ago reported the period during which she made use of alternate healing systems: *"I did feel better after the treatment but in the middle I became very sick. When my condition worsened… my mother took me to a traditional healer.... who boiled some herbs for me to drink..... Then my mother heard about a witchdoctor who said he would be able to help me. He also gave me herbal medication to drink. It didn’t really help, but I continued to use it with the hope that I was going to get better. But I noticed that since I started using the medication from the health center, my health has improved a lot."*

#### Delay in diagnosis by health care providers

A few of the TB patients who visited health care facilities reported that health care providers failed to suspect and diagnose TB early in the course of their illness. As it was observed, the health centers in the study area were not well-equipped with TB diagnostics, and their staff were not well trained on early diagnosis and DOTS treatment of patients presenting with symptoms of TB. This resulted in unnecessary trips to health facilities, delayed diagnosis and care, leading to an inefficient uses of scarce financial resources by patients. A 39-year-old male patient who took 12 months to be enrolled in the treatment program reported a long delay in diagnosis in the following ways:

*"I went to the nearby health center two times … I was told I had pneumonia … then to the nearest hospital … the doctor told me to go for x-ray and VCT… then I was found to have TB."* Various reasons for delaying tuberculosis treatment are summarized in Table 1.

**Table 1:** Reasons for delay in tuberculosis diagnosis in Dabat, Ethiopia, 2011

**Reason for delay**	(Number of participants)*
Lack of geographic access to health facilities	(20)
Financial burden	(17)
Use of traditional healing system	(12)
Delay in diagnosis by health care providers	(5)

* Numbers in parentheses show the number of patients who reported the reasons. They do not add up to 26 as some patients reported more than one reason.

### Theme two: Reasons for tuberculosis treatment non-compliance

#### Geographic access to health facilities

All the participants reported that the first two months of treatment were physically exhausting. This was particularly the case for the many patients who were at advanced stages of the disease at the time of starting treatment. Patients attending treatment from urban kebeles had shorter walking distances than those from rural kebeles. Most patients from rural kebeles reported travelling one to ten hours single trip. A 20-year-old male patient with advanced symptoms, who was also suffering from various side effects, described his daily walk to the health centre as follows:

"Well, I didn’t have a problem with taking treatment. The only problem I had was coming for treatment by walking long distance to reach the TB treatment center. I was more challenged during harvest."

Travelling long distance to the health care facility with costly payment competed with other essential expenses and challenged patients’ capacity to collect their drugs. Some patients overcame the problem by borrowing money from friends, according to a male patient aged 45 years:

"We have no choice but to come to this health center every day, particularly during the intensive phases of treatment to get the medicine. What is challenging is the transportation expense. However, I couldn’t give up because it is a matter of life."

#### Financial burden

The majority of TB patients confirmed that limited access to money was the main reason for treatment non-compliance. At the intensive phase of the treatment, TB patients were required to take their treatment under a direct observation of health personnel; as a result, patients who came from distant areas needed to rent houses in the towns where TB treatment centers were located and had to pay for food, transportation to receive treatment, medical examinations and drugs, hospitalization, and expensive referrals to other health care facilities. The overall life conditions of most TB patients as was explained during the interview were unbearable as many of them had severe financial constraints. Patients’ resignation, helplessness, loss of social independence, and lack of alternatives were often associated with their inability to work or otherwise provide for their needs. Patients who faced such challenges prefer to interrupt treatment despite their desire to continue. What participants said about the financial challenges they faced was narrated as follows by a 45-year-old female patient with TB-HIV co-infection:

"My husband passed away recently. To survive, I had to take medicine. Currently I have lots of stress because of the financial difficulties. ‘I am not working……my children are growing, and they have increasing needs which I cannot meet. It is a heavy burden on me."

A male patient aged 42 years said: *"One of the challenges I have faced is inability to get food. At the health center, the nurses tell us that we need to eat more good food, but the money is not there to buy such things.”* Sometimes "*It has been very hard for me to take treatment because I don’t have anything to eat. There were days when I begged on the roads just to collect money to buy something to eat so that I could continue taking my treatment."*

#### Quality of health service provided

Weight gained during treatment and receiving improved laboratory results were reported to enhance compliance. Actual events of adverse drug reactions and fear of possible adverse drug reactions reduced treatment compliance. Overcoming these difficulties was best accomplished with support from service providers. While interaction based on trust between patients and care providers was expressed as essential for a successful completion, the patients could easily lose their trust in DOTS if they felt that the providers did not take their concerns seriously.

When TB patients were asked about their perception of the quality of the health care they received, their responses clustered around one theme, namely the attitude of the health care team at the health centers in the study area. Most of them were satisfied with the facilities available and the interaction they had with certain staff. The comments of a male and a female patients 50 and 25 years old, respectively, follows:

"The assistance I received at the TB clinic was very good. The nurse understood and did the best she could for me. Sometimes even when I had another problem, she was willing to listen and do follow ups."

"My experience at this DOTS Clinic is good. I find the nurse good at her job. She is very helpful, especially Sister [name]. I have no complaints, honestly. This is where I have found real help."

#### Dynamics of social support

The support of families and the community was extremely important during the intensive phases of treatment, because this to a large extent compensates for loss of income. Even though TB causes fear and stigma, people share resources in times of crisis, and reciprocal arrangements provide most TB patients with some food. Many patients receive support from families and close friends either in kind or money for transportation. How family support can influence treatment compliance is narrated as follows by a male patient aged 27 years:

"Recently, I completed my treatment because of my family members’ strong support….They encouraged me to take the drug."

A 23-year-old male patient said*:*"*Even though I couldn’t get good food, I managed to get as much food as possible. My family, relatives, and neighbors provided me with enough food."*

The other 39- year-old female patient said*:"I stopped taking my drug because of squabbles in my family … Hostility among family members can discourage one from taking drugs."* Table 2 shows various reasons for tuberculosis treatment non-compliance.

**Table 2:** Reasons for tuberculosis TB treatment non-compliance in Dabat, Ethiopia, 2011

**Reasons for non-compliance**	(Number of participants)*
Lack of geographic access to health facilities	(26)
Financial burden	(15)
Quality of health services provided	(9)
Dynamics of social support	(7)

* Numbers in parentheses show the number of patients who reported the reasons. They do not add up to 26 as some patients reported more than one reason.

## Discussion

This study suggests that TB patients have to overcome great challenges in seeking care early and complying with TB treatment. The majority of the TB patients reported that difficulty of geographic access to health facilities, financial burden, use of traditional healing system and delay in diagnosis by health care providers were the main reasons for not initiating TB treatment timely. Problem of geographic access to health facilities, financial burden, quality of health services provided and social support were identified as the main reasons for failing to fully comply with TB treatment.

Problems of physical access to TB diagnosis and treatment centres were the most important factors influencing early TB treatment initiation and compliance [12,13]. We found that many patients still experience difficulties related to traveling to health facilities for diagnosis and daily attendance for treatment. There were only two health centers in the study area providing health services including DOTS to a population of over 145,458 [29]. The majority of the people in these settings lived in areas which required more than two hours to reach a health facility. In an effort to reduce the access gap to health care, the Ethiopian Government had started a new community-based initiative called the “Health Service Extension Program”. Under this program, two health extension agents were identified and trained in each kebele (smallest administrative unit in Ethiopia with an estimated population of 5000) to enhance case holding under the DOTS program through the decentralization of services in the country [13,30]. The provision of transport fee and permitting some of the poorest or the most ill patients to take medicine home for self-treatment may be a means to increase treatment adherence. A recent review of randomized control trials, comparing DOTs with self-administration of therapy, provided no evidence that self-administration of anti-TB in low-and middle-income countries reduced cure or treatment completion problems in people with TB [31]. Several authors have advocated a shift in perspective where patients' socioeconomic environments, their well-being, and dignity are considered in future strategies. Strategies based on self-treatment can be strengthened by support and supervision by identified relatives, neighbors, or through other social structures like the ‘Idir’, which is a community-based body with social responsibility mainly for arranging funerals, or ‘TB clubs’, which are small groups of patients who live near each other [32,33]. Several TB control programmes that leave the choice of DOT supervisor to the patient have been shown to be successful [34].

The financial burden on TB patients was one of the key issues influencing timely treatment initiation and compliance in our study. In Ethiopia, TB diagnosis and treatment are meant to be provided free of charge with the aim of decreasing the financial burden on patients [35]. However, the main financial burdens, as evidenced in the present study, are the extra costs of transportation, medical examinations, the need to purchase liver protection drugs, hospitalization costs, and expenses of basic necessity. Moreover, the needed ancillary treatment is charged with significant financial burden on patients and their families. The brutal level of poverty that the participants of this study faced often meant that they were living below the breadline, unable to meet their own and their families’ basic needs for health care. Thus, improving access to DOTS including the required ancillary treatment services can significantly reduce financial burden on TB patients.

The use of an indigenous local healing system was also reported by most participants in this study. Prolonged self-treatment involving traditional healing systems are the first step in the health-seeking behavior process. In developing countries, it is estimated that about half the general public uses complementary and alternative medicine [7,18,19]. In this study, we understood that such practices are common in areas where TB patients first try local healing practice before turning to DOTS services. This finding is consistent with previous studies and is linked to failure to identify TB suspects [17].There is a need for interventions that encourage symptomatic individuals to seek modern medical care as early as possible by establishing links to alternative health care providers.

The inability of health services to suspect and diagnose TB patients at first contacts contributed to late TB treatment initiations. In this study, a few of the TB patients who visited health care facilities reported that health care providers failed to suspect and diagnose TB early enough in the course of their illness. As it was observed, the health centers in the study area were not well-equipped with TB diagnostics. This confirmed findings from previous studies that showed that health units failed to investigate chronic coughs in a certain proportion of TB suspects [3,6,17]. This finding raises several programmatic and policy implications. Misdiagnosis and faulty treatment lead the patient to loss of scarce time and money in search of treatment and may increase the duration of illness and the possibility of death. For public health officials, misdiagnosis results in the underestimation of TB incidence rates and increases the duration of infectivity in the community. Interventions like the implementation of standard screening procedures and rapid diagnostics, training and close supervision of health care providers, strengthening quality control and education of patients so that they expect and request diagnostic testing for TB when appropriate, and reduced costs for diagnostic tests could improve the likelihood of TB diagnosis at health facilities.

The quality of health care participants received was an important factor that influenced treatment compliance. The relationship between health care practitioners and participants is indicative of a good “therapeutic alliance”, a process in which the practitioner effectively understands the patient’s problem and formulates a management plan that is conducive to compliance. A good therapeutic alliance is underpinned by trust, empathy, and positive regard [7,21]. Participants seemed to suggest that good communication and involvement in the treatment process equipped TB patients with the capacity to play active roles in managing their own health. This finding is consistent with that of other studies [22,23]. An improvement in these aspects of TB treatment is crucial in encouraging patients to continue with treatment for the full duration of the regimen.

In this study, family support is identified as the most important factor for influencing treatment compliance. It is possible that family support can alleviate patients’ economic and social problems. Family members can also observe patient adherence to medications, provide encouragement, and remind them of their medical appointments [12,24,25]. A previous study from Ethiopia found that most patients interrupted medication in their third or fourth month of treatment [13] which may indicate fading family support during the later phases of treatment [26].

The validity of the findings of this study was strengthened by the triangulation of the source, that is collecting data from patients who were on treatment and who had interrupted treatment. Additionally, the validity of the study should have further been strengthened by triangulation of researchers, that is by involving multiple researchers to analyze the data, develop and test the coding scheme. It is also expected to have ensured a wider representation of experiences and views on the subject matter making it relevant to other rural settings in Ethiopia and elsewhere in Sub-Saharan African countries.

## Conclusions

This study highlighted complexities surrounding TB control efforts in Dabat District. Challenges of geographic access to health care facilities and financial burden were factors that most influenced timely TB treatment initiation and compliance. Decentralization of TB diagnosis and treatment services to peripheral health facilities, including health posts is of vital importance to make progress toward achieving TB control targets in Ethiopia.

## Competing interests

The authors declared that they have no competing interests.

## Authors’ contributions

TT initiated the research, wrote the research proposal, conducted the research, did data analysis and wrote the manuscript. MD, YB, YK and MA involved in the write up of the proposal, data analysis, and write up of the manuscript. All authors read and approved the final manuscript.

## Pre-publication history

The pre-publication history for this paper can be accessed here:

http://www.biomedcentral.com/1471-2458/13/424/prepub
